# Microfluidic cell engineering on high-density microelectrode arrays for assessing structure-function relationships in living neuronal networks

**DOI:** 10.3389/fnins.2022.943310

**Published:** 2023-01-09

**Authors:** Yuya Sato, Hideaki Yamamoto, Hideyuki Kato, Takashi Tanii, Shigeo Sato, Ayumi Hirano-Iwata

**Affiliations:** ^1^Research Institute of Electrical Communication, Tohoku University, Sendai, Japan; ^2^Graduate School of Biomedical Engineering, Tohoku University, Sendai, Japan; ^3^Faculty of Science and Technology, Oita University, Oita, Japan; ^4^Faculty of Science and Engineering, Waseda University, Tokyo, Japan; ^5^Advanced Institute for Materials Research, Tohoku University, Sendai, Japan

**Keywords:** microfluidic devices, microelectrode array (MEA), complex networks, cultured neuronal network, cell engineering

## Abstract

Neuronal networks in dissociated culture combined with cell engineering technology offer a pivotal platform to constructively explore the relationship between structure and function in living neuronal networks. Here, we fabricated defined neuronal networks possessing a modular architecture on high-density microelectrode arrays (HD-MEAs), a state-of-the-art electrophysiological tool for recording neural activity with high spatial and temporal resolutions. We first established a surface coating protocol using a cell-permissive hydrogel to stably attach a polydimethylsiloxane microfluidic film on the HD-MEA. We then recorded the spontaneous neural activity of the engineered neuronal network, which revealed an important portrait of the engineered neuronal network–modular architecture enhances functional complexity by reducing the excessive neural correlation between spatially segregated modules. The results of this study highlight the impact of HD-MEA recordings combined with cell engineering technologies as a novel tool in neuroscience to constructively assess the structure-function relationships in neuronal networks.

## Introduction

Connectomics analyses provided anatomical information of the nervous system of animals at resolutions ranging from individual cells to brain regions (Gao et al., 2019). The studies have revealed several non-random properties such as the modular architecture as a structure that is evolutionarily conserved in the nervous system (van den Heuvel et al., 2016) and provided mechanistic insights into how network structure defines system functions in both normal and pathological brains (Lynn and Bassett, 2019; van den Heuvel and Sporns, 2019; Suárez et al., 2021). While many studies have deciphered the structure-function relationships in the nervous system *in vivo* (Meunier et al., 2010; Lee et al., 2016), recent advances in cell engineering technology using micropatterned proteins and microfluidic devices have enabled the use of cultured cells to study these relationships in a well-defined *in vitro* system (Feinerman et al., 2008; Lewandowska et al., 2015; Pan et al., 2015; Albers and Offenhäusser, 2016; Yamamoto et al., 2016b,^2018^; Forró et al., 2018; Hong and Nam, 2020, 2022; Takemuro et al., 2020; Duru et al., 2022; Ihle et al., 2022).

The two major technologies employed to record network activities in cultured neurons are fluorescence calcium imaging, which offers advantages in spatial resolution, and microelectrode arrays (MEA), which offer advantages in temporal resolution. Recently, high-density MEA (HD-MEA) technology has been developed to mitigate the trade-off problem between spatial and temporal resolutions (Berdondini et al., 2009; Frey et al., 2010; Hierlemann et al., 2011; Bertotti et al., 2014; Obien et al., 2015; Yuan et al., 2020; Steinmetz et al., 2021). More precisely, recent HD-MEA devices offer spatial resolutions of over 3,000 electrodes mm^–2^ with the electrode pitch below 20 μm and a temporal resolution below 100 μs (Bertotti et al., 2014; Lewandowska et al., 2015; Kim et al., 2020; Yuan et al., 2020; Steinmetz et al., 2021; Duru et al., 2022; Shimba et al., 2022). The electrode pitch is comparable to the size of a neuronal cell body, and the temporal resolution is higher than a typical delay of synaptic transmission (∼0.6 ms) (Lisman et al., 2007). Thus, the combination of the cell engineering and HD-MEA technologies provides a new framework to assess structure-function relationships in biological neuronal networks with unprecedented spatial and temporal resolutions.

Here, we fabricated neuronal networks possessing a modular architecture on HD-MEA using a polydimethylsiloxane (PDMS) microfluidic film and recorded their spontaneous activity at a resolution of 50 μs. Engineering neuronal networks of HD-MEA has been challenging due to the surface topography originating in the passivation layer and the underlying electronics of the device (Frey et al., 2010; Hierlemann et al., 2011), which inhibits stable sealing of microfluidic devices (Duru et al., 2022). We resolved this issue by coating the HD-MEA surface with a cell-permissive hydrogel which smoothened the surface topography of the HD-MEA, enabling a gap-less adhesion of the PDMS microfluidic film to the HD-MEA. Recordings of neural activity at high temporal resolution revealed that modular architecture suppresses excessive neural correlation between spatially segregated modules and enhances functional complexity of the network. Correlation coefficients were used to assess the degree of synchrony between two electrodes, while functional complexity was used to quantify the degree of integration-segregation balance in each network (Zamora-López et al., 2016). Furthermore, functional modularity was calculated from correlation matrices to evaluate the degree of modularization in a network. Finally, we evaluated the spatiotemporal structure of the network dynamics by analyzing the statistics of neuronal avalanches (Beggs and Plenz, 2003; Plenz and Thiagarajan, 2007). Our results highlight the impact of HD-MEA recordings combined with cell engineering technologies as a tool to assess the structure-function relationships in neuronal networks.

## Materials and methods

### Microelectrode array and hydrogel coating

MaxOne HD-MEA chips (MaxWell Biosystems), bearing 26,400 electrodes with an interelectrode separation of 17.5 μm, were used in this study. The HD-MEA chip, as received, was first exposed to air plasma (Yamato PM100) for 60 s to hydrophilize the electrode area. The chip was then sterilized in 70% ethanol for 30 min and subsequently rinsed in sterile deionized water three times.

The electrode area was then coated by a cell-permissive hydrogel and poly-D-lysine (PDL). The hydrogel layer was formed by drop casting 50 μl of collagen solution [1:1 mixture of type-I collagen solution (5 mg ml^–1^, Koken AteloCell IAC-50) and Neurobasal medium (Gibco 21103-049) supplemented with 2% B-27 (Gibco 17504-044) and 1% GlutaMAX-I (Gibco 35050-061)] on the electrode area of approximately 2.1 × 3.9 mm^2^, removing excessive volume of the solution with a micropipette, and then inducing gelification of the remaining solution in an CO_2_ incubator (37°C) overnight and in a refrigerator (4°C) overnight. The sample was then dried in a clean bench overnight to complete vitrification (Takezawa et al., 2004; Matsumura et al., 2016). PDL solution [50 μg ml^–1^ PDL (Sigma P-0899) in Dulbecco’s PBS] was then drop-casted onto the electrode area coated with the hydrogel. After 1 h, the PDL solution was aspirated, and the chip was rinsed in deionized water three times. Finally, the chip was dried and stored at room temperature until use. The surface topography of the HD-MEA and the hydrogel layer was analyzed by confocal microscopy (Keyence VK-X260).

### Microfluidic device

PDMS microfluidic films for cell patterning were fabricated as detailed previously (Takemuro et al., 2020). Briefly, the master mold was fabricated by patterning two layers of SU-8, i.e., SU-8 3005 (3,000 rpm, 60 s; ∼5 μm) and SU-8 3050 (1,500 rpm, 60 s; ∼100 μm), via photolithography. The geometry of the microchannels was defined by the first layer, whereas the geometry of the square through-holes was defined by the second layer. The microfluidic film was then fabricated by drop casting Sylgard 184 (Dow Corning; ratio, 7.5:1) onto the master mold and thermally curing it at 70°C for 2 h. Two types of micropatterned neuronal networks were fabricated, i.e., “random” and “modular” networks. The random network comprised an isolated square through-holes of 400 × 400 μm^2^, whereas the modular network comprised four square through-holes of 200 × 200 μm^2^ connected neighbor to neighbor by microchannels. The fabricated microfluidic device was gently removed from the master mold using forceps and cut in a size of approximately 3 × 4 mm^2^ using a sharp knife. The device was then cleaned and sterilized by sonication in 100% ethanol, rinsed in deionized water three times, dried in air, and further sterilized under UV-light for 30 min. Finally, the device was placed on the hydrogel-coated electrodes using forceps.

### Cell culture

Primary cortical neurons were obtained from the cerebral cortices of embryonic day 18 rats. First, the cerebral cortices were collected from rat embryos and placed in a 60-mm dish containing 4.5 ml of Hank’s balanced salt solution (HBSS; Gibco 14175-095) supplemented with 10 mM HEPES (Gibco 15030-015) and 1% penicillin/streptomycin (Sigma P-4333). After cutting the tissue into small pieces of approximately 1 mm^3^, the tissue and HBSS were transferred to a 15-ml centrifuge tube. The tube was then supplemented with 0.5 ml of 2.5% trypsin (Gibco15090-046; final concentration, 0.25%) and 0.2 ml of 10 mg/ml DNase (Roche 10104159001; final concentration, 0.4 mg/ml) and incubated at 37°C for 15 min. After the incubation, all excess solution was aspirated, and the tissue was rinsed three times with fresh HBSS. The tissue was then triturated with fire polished glass pipettes to disperse the cells.

Prior to culturing the rat cortical neurons, the well of the HD-MEA chip was filled with the neuronal plating medium [minimum essential medium (Gibco 11095-080) supplemented with 5% fetal bovine serum (Gibco 12483) and 0.55% glucose (Sigma G-8769)], and the chip was incubated in a CO_2_ incubator (37°C, 5% CO_2_) for at least 3 h. Cortical neurons were then plated at a density of 5.3 × 10^4^ cells/cm^2^. After 1 h, the entire medium was replaced with the Neurobasal medium supplemented with 2% B-27 and 1% GlutaMAX-I, and half the medium was replaced with fresh Neurobasal medium twice every week.

### Data recording and analysis

Spontaneous neural activity was recorded using the MaxLab Live software (MaxWell Biosystems) at a sampling frequency of 20 kHz for 30 min. Electrodes used for recording were selected by scanning the activity of each electrode located in the through-holes and finding “active electrodes,” i.e., electrodes with a firing rate over 0.02 Hz and a signal amplitude above a threshold. Each chip contained eight independent networks, and the average number of active electrodes per network was 85.9 ± 23.7 (*n* = 31 networks).

Action potentials were detected from high-pass filtered (> 300 Hz) signal with a threshold of –5 × SD. As the objective of the work was to elucidate the functional consequence of the topological control in cultured neuronal networks, spike sorting was not performed to separate spike trains of single neurons. The recorded voltage traces and scatter plots was grouped into modules using a custom MATLAB script. Pairwise correlation between electrodes *i–j*, *r*_*ij*_, was calculated as $r_{i\,j}=\frac{\text{Cov}\,\left(n_{i},n_{j}\right)}{\sqrt{\text{Var}\,\left(n_{i}\right)\,\text{Var}\,\left(n_{j}\right)}}$, where *i* and *j* are electrode indices, *n_*i*_ = n_*i*_*(*t*) is the spike train of electrode *i*, and Cov and Var are the covariance and variance over the entire time bins, respectively. The bin width was set to 50 ms, and *n*_*i*_(*t*) was one if the electrode detected more than one spikes in the *t*-th time bin, and zero otherwise. Definition and calculation of other statistical measures are described in the corresponding sections below.

From the correlation matrices, functional complexity *C* (Zamora-López et al., 2016; Yamamoto et al., 2018) was evaluated as:


(1) $$C=1-\frac{1}{C_{m}}\,\sum_{\mu =1}^{m}\left|p_{\mu }\,\left(r_{i\,j}\right)-\frac{1}{m}\right|$$

where *p*_μ_ (*r*_*ij*_) is the probability of *r*_*ij*_ to be in the μ-th bin, *C*_*m*_=2(*m*−1)/*m* is a normalization factor, and *m* = 20. For the statistical analysis of the functional complexity, electrodes with a mean firing rate below 0.5 Hz were excluded from the analysis as non-active electrodes, and networks with more than 20 active electrodes were used.

The degree of modularization in the functional connectivity of the networks was also quantified from the correlation matrices by calculating modularity *Q* of the matrix (Newman, 2006):


(2) $$Q=\frac{1}{2\,E}\,\sum_{i=1}^{N}\sum_{j=1}^{N}\left(A_{i\,j}-\frac{k_{i}\,k_{j}}{2\,E}\right)\,\delta_{m_{i}\,m_{j}}$$

where *A* = [*A*_*ij*_] is a binarized correlation matrix generated by thresholding *r*_*ij*_ at 0.7, 2*E* = ∑_*ij*_*A*_*ij*_ is the total number of edges (*A*_*ij*_ = 1) in the matrix, *k*_*i*_ = ∑_*j*_*A*_*ij*_ is the node degree of *i*, *δ_*mimj*_* is the Kronecker delta function which is equal to one if nodes *i* and *j* belong to the same module (*m*_*i*_ = *m*_*j*_) and zero otherwise. A positive value of *Q* indicates the presence of modular structure with a maximum of *Q* = 1. In contrast, *Q* = 0 if the matrix lacks modular structure and is random.

Neuronal avalanche statistics was computed on the local field potential (LFP) signals that were detected simultaneously with the spiking activity. More precisely, LFP was extracted by down-sampling the recorded voltage traces to 1-ms resolutions and band-pass filtering at 1-100 Hz, and a negative peak deflection in the LFP (nLFP) was detected at each electrode by thresholding the LFP signal at –3 × SD (Beggs and Plenz, 2003). The nLFP raster was then binned at 4 ms, and avalanches were detected as a series of bins between two empty bins. The avalanche size was then evaluated as number of nLFPs in an avalanche, and the avalanche duration as the number of consecutive bins with more than one nLFP multiplied by 4 ms. Finally, the distribution of avalanche sizes and durations were plotted as probability histograms in double logarithmic axes.

### Statistical analysis

Data are presented as mean ± standard deviation (SD). Student’s *t*-test and chi square test were used as described in the respective sections. The significance level was set to *p* < 0.05. The statistical analyses were performed on MATLAB R2020a or Microsoft Excel.

## Results

### Hydrogel coating of HD-MEA

**FIGURE 1:**
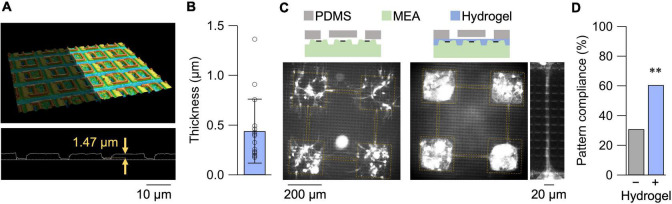
Hydrogel coating of HD-MEA. **(A)** 3D confocal micrograph of the HD-MEA chip (top) and its surface profile (bottom). **(B)** Thickness of the hydrogel films in their dried state measured by confocal microscopy. Data shown as mean ± SD, with plots for individual data points. **(C)** Primary neurons cultured on HD-MEA chip without (left) and with (right) the hydrogel coating. A magnified view of the microchannel region of a separate sample is also presented for the hydrogel-coated chip. The cells were stained with Neu-O (Er et al., 2015), and yellow dashed lines depict presumptive locations of microfluidic structures. **(D)** Fraction of compliant patterns with and without the hydrogel coating. ^**^p < 0.01 (chi square test).

A confocal micrograph of the HD-MEA and its surface profile is shown in Figure 1A. The depth of the surface groove was approximately 1.5 μm, greater than the diameter of axons and dendrites (Bartlett and Banker, 1984). We, thus, coated the HD-MEA surface with a collagen hydrogel layer with a thickness of 0.44 ± 0.32 μm in its dried state (mean ± SD; *n* = 16 measurements from 8 samples in 3 preparations; Figure 1B). With the reported swelling ratio of approximately five (Kim and Kim, 2016), the hydrogel layer was sufficient to coat the surface topography of the HD-MEA. Primary neurons cultured on the HD-MEA with a PDMS microfluidic film placed on top are shown in Figure 1C. Without the hydrogel layer, non-specific growth of neurites was observed due to the space that remained at the base of the PDMS microfluidic film. Surface treatment with the hydrogel suppressed the non-specific neurite outgrowth, and the fraction of compliant networks, i.e., patterns without non-specific neurite growth, increased from 0.25 (*n* = 8 networks) to 0.67 (*n* = 32 networks; Figure 1D).

**FIGURE 2:**
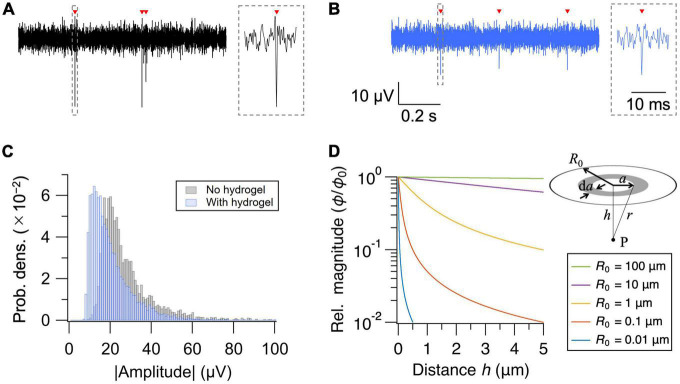
Effect of hydrogel coating on signal amplitude. **(A,B)** Recorded extracellular signals (high-pass filtered at 300 Hz) from representative electrodes without **(A)** and with **(B)** the hydrogel coating. Detected action potentials are indicated with red arrowheads. Close-up views of the waveforms are shown in the insets. **(C)** Distribution of the mean signal amplitude for each electrode. **(D)** Relative magnitude of the electric potential (ϕ/ϕ_0_) as a function of distance (*h*) from the current source. Distance dependent decay depends strongly on the diameter (*R*_0_) of the current source.

Insertion of the hydrogel layer increases the cell-electrode distance. We, therefore, assessed how much the hydrogel coating degrades the signal amplitude of the extracellular action potentials. Representative waveforms of extracellular action potentials recorded without and with the hydrogel layer are shown in Figures 2A, B, respectively. Presence of the hydrogel layer decreased the median signal amplitude by 29%, from 23.2 to 16.5 μV (Figure 2C). The signal amplitude, however, remained well above the noise level (<5 μV_*rms*_) of the current MEA setup, securing the use of hydrogel coating as a novel approach to interface PDMS microfluidic device and HD-MEA.

Although the cell-electrode configuration in the current experiment seemingly contradicts with the classic point-contact model of extracellular recordings, non-contact recordings of extracellular action potentials have previously been demonstrated in cardiomyocytes (Sharf et al., 2019), and their mechanism can be described by the volume conductor theory (Obien et al., 2015). Briefly, if we model a neuron as a two-dimensional disk in which current sources with current density *j*_0_ are uniformly distributed, the electric potential ϕ at some point P on the axis of symmetry perpendicular to the disk is given by:


(3) $$\phi =\frac{j_{0}}{2\,\sigma }\,\left(\sqrt{h^{2}+R_{0}^{2}}-h\right)$$

where *h* (> 0) is the distance between the center of the disk to point P, *R*_0_ is the radius of the disk, and σ is the conductivity of the environment (Nunez and Srinivasan, 2009; Purcell and Morin, 2013; Sharf et al., 2019). If we write the electric potential at *h* = 0 as ϕ_0_, the relative magnitude of the potential at distance *h* is given by:


(4) $$\phi /\phi_{0}=\frac{\sqrt{h^{2}+R_{0}^{2}}-h}{R_{0}}$$

Dependence of ϕ/ϕ_0_ on *h* is plotted in Figure 2D for various *R_0_*. Although electric potential rapidly decreases with increasing distance for small current sources, the dependence of the magnitude on distance becomes weaker with increasing size of the current source. If we assume that the thickness of the hydrogel layer is equal to the neuron-electrode distance and solve for *R*_0_ that gives –29% decrease in signal amplitude (ϕ/ϕ_0_=0.71) at *h* = 2.2 μm (=0.44 × 5), we obtain *R*_0_ = 6.3 μm, which is consistent with the typical size of a neuronal cell body. Therefore, while it is inevitable that the insertion of the hydrogel layer decreases the signal amplitude, the extracellular recording of action potentials from cells separated by a few μm is physically reasonable.

### Spontaneous activity in engineered neuronal networks

**FIGURE 3:**
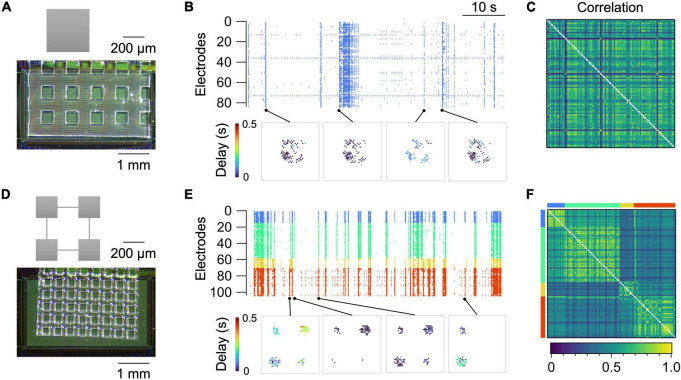
Spontaneous activity in random **(A–C)** and modular **(D–F)** networks at 14 DIV. **(A,D)** Micropattern geometry and a micrograph of the PDMS microfluidic film placed on the sensing electrode area of the HD-MEA chip. **(B,E)** Raster plot (top) and representative propagation maps (bottom). A propagation map for a given network burst was generated by setting a marker at the position of electrodes that were active during the burst and coloring the markers based on the time delay from the onset of the burst. **(C,F)** Correlation coefficient matrix. For the modular network, electrodes belonging to separate modules are assigned different colors in **(E,F)**.

Figure 3 summarizes representative recordings of spontaneous neural activity from two types of micropatterned neuronal networks, i.e., a “random” network and a “modular” network. The random network was an isolated square of 400 × 400 μm^2^, in which neurons grew uniformly and formed random connections (Figure 3A). The size was chosen so that each network stably generates spontaneous activity after approximately 10 days of culture (Yamamoto et al., 2016a). The activity of the network was predominantly governed by the network bursts, i.e., a population activity that entrained a large fraction of the network (Figure 3B; Orlandi et al., 2013; Yamamoto et al., 2016a). This caused the pairwise correlation coefficient *r*_*ij*_ to be high between a large fraction of electrode pairs (Figure 3C).

Modular networks, in contrast, exhibited a richer repertoire of population activity. Modular networks comprised four squares, or modules, of 200 × 200 μm^2^ connected by microchannels with widths and heights of 6.7 ± 0.79 μm (*n* = 12) and 4.3 ± 0.05 μm (*n* = 10), respectively (Figure 3D). The microchannel allowed axons and dendrites of a fraction of neurons to project to neighboring modules and form functional couplings between them (Takemuro et al., 2020). While activity of the neurons in the same module was strongly correlated, the interaction of neurons in separate modules was weaker, leading to probabilistic coherence (Figures 3E, F). Cross-correlation functions between pairs of electrodes in the same module exhibited a large peak around a lag of zero, whereas the peak was much smaller for electrode pairs in separate modules. This clearly contrasted with the cross-correlation functions in a random network, where the electrode-to-electrode difference in the shape of the function was less pronounced (Supplementary Figure 1).

Detailed comparisons of activity statistics in the random and modular networks are summarized in Supplementary Figure 2. Network bursts were also observed occasionally in modular networks. Within a network burst, the timing of neuronal spikes in a single module was much less varied than that across separate modules (Supplementary Figure 3A). The high spatiotemporal resolution of the HD-MEA, however, allowed us to capture the propagation of the activity even inside a single module (Supplementary Figure 3B). Recordings of spontaneous activity could also be obtained from more mature cultures, which we confirmed at least up to 21 DIV (Supplementary Figure 4).

### Functional complexity and modularity

**FIGURE 4:**
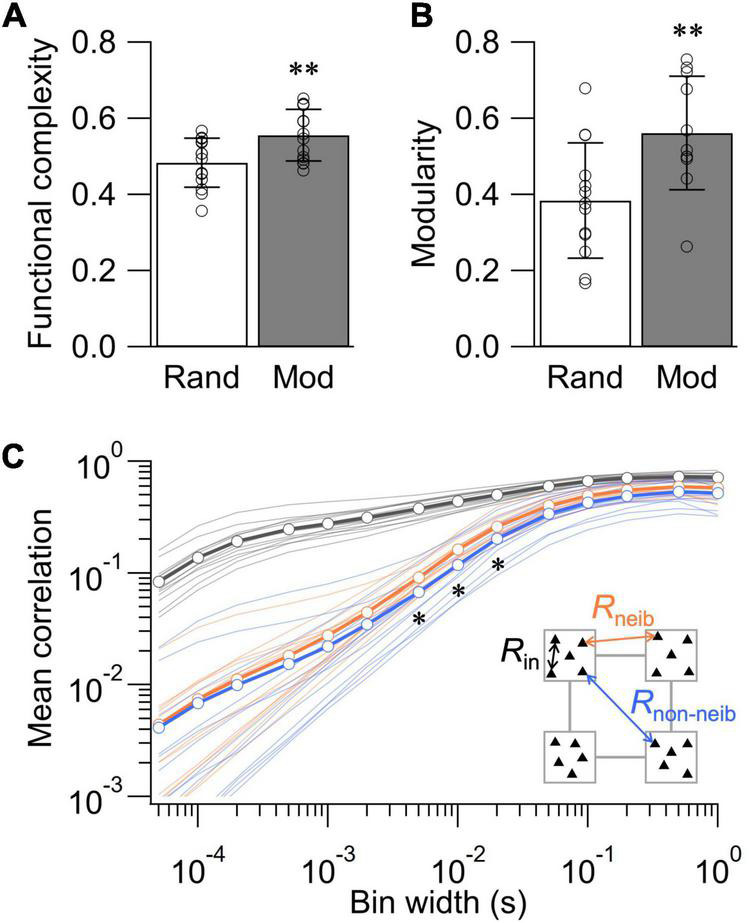
Structure-function relationships in cultured neuronal networks. **(A)** Functional complexity for the random and modular networks. **(B)** Modularity of binarized correlation matrix for the random and modular networks. Data shown as mean ± SD, with plots for individual data points. **(C)** Dependence of mean correlation coefficient on the bin width of the spike train. *R*_in_, correlation between electrodes in the same module; *R*_neib_, correlation between electrodes in neighboring modules; *R*_non–neib_, correlation between electrodes in non-neighboring (diagonal) modules. *p < 0.05, ^**^p < 0.01 (two-tailed *t*-test).

To statistically evaluate the impact of network structure on the dynamics of spontaneous activity, we further assessed functional complexity, functional modularity, and the statistics of *r*_*ij*_ and neuronal avalanches in the random and modular networks. Functional complexity *C* is a measure of integration-segregation balance important for proper functioning of the nervous system, with *C* = 1 and 0 indicating maximally and minimally balanced states, respectively (Zamora-López et al., 2016; Yamamoto et al., 2018). Previous studies using calcium imaging have shown that patterning cortical neurons in modular architecture broadens the distribution of *r*_*ij*_ and increases the value of *C* (Yamamoto et al., 2018; Takemuro et al., 2020). Analyses of the spontaneous neural activity recorded at 10–14 DIV revealed that the value of *C* was significantly higher in the modular network than in the random network, confirming the previous observations (Figure 4A; *p* < 0.01, *n* = 14 for both random and modular networks; two-tailed *t*-test). The analysis of Newman modularity *Q* (Newman, 2006) in the correlation matrices further revealed that the functional connectivity is more modularized in the modular network (Figure 4B; *p* < 0.01, *n* = 13 and 11 for random and modular networks, respectively; two-tailed *t*-test). These data highlight that structural confinement induced functional modularization in the cultured neuronal network.

We next took the advantage of high temporal resolution of MEA recordings to assess how the mean correlation coefficient *R* = ∑_*ij*(*i*≠*j*)_*r*_*ij*_/(*N*^2^−*N*)of modular networks depends on the temporal bin width (Figure 4C). To this end, we classified *R* into three categories: (1) *R*_in_, the mean value of *r*_*ij*_ evaluated within each module; (2) *R*_neib_, the mean evaluated across neighboring modules; and (3) *R*_non–neib_, the mean evaluated across non-neighboring modules. As a general trend, the values of *R* were dependent on the bin width of the spike train and increased when a larger bin was used. Comparison at a constant bin width revealed that of *R*_in_ was larger than *R*_neib_ and *R*_non–neib_ at any bin width, indicating that intramodular correlations were greater than intermodular ones. The difference between *R*_neib_ and *R*_non–neib_ was smaller than that against *R*_in_. However, *R*_non–neib_ was significantly smaller than *R*_neib_ for the bin width between 5 and 20 ms (*p* < 0.05; *n* = 14 networks; two-tailed *t*-test). The lower value of *R*_non–neib_ is expected as non-neighboring modules are spatially more distanced than neighboring modules, increasing the signal delay via signal propagation and synaptic transmission. Importantly, such analysis was impossible with low-temporal resolution recordings of neural activity and highlights a novel potential of MEA recordings in the assessment of structure-function relationships in living neuronal networks.

### Neuronal avalanche statistics

**FIGURE 5:**
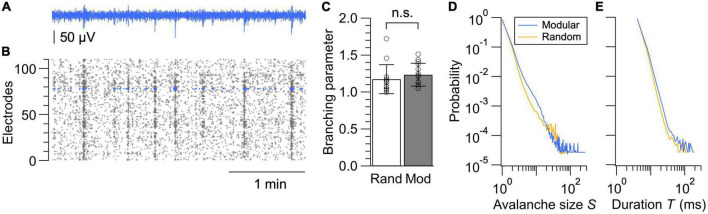
Neuronal avalanche analysis. **(A)** LFP signal from a representative electrode. **(B)** nLFPs extracted from all electrodes in a modular network. Marker sizes are varied proportional to their amplitudes. **(C)** Branching parameters calculated from the random and modular networks. Data shown as mean ± SD, with plots for individual data points. n.s., no significance (two-tailed *t*-test). **(D,E)** Size distributions **(D)** and duration distributions **(E)** of neuronal avalanches in random and modular networks. Networks with sufficient activity were selected based on the same criteria used for the analysis in Figure 4A, and average probability distributions calculated from 14 networks are shown for both the random and modular networks.

Finally, we compared the spatiotemporal structure of the spontaneous neural dynamics in random and modular networks by analyzing the neuronal avalanches in LFP signals (Figure 5A). Neuronal avalanche is defined as a sequence of time bins during which an nLFP was detected in at least one electrode. An nLFP reflects the summation of local inward currents, and its analysis uncovers whether the system operates near a critical point, at which activity stably propagates and maximizes information transmission within a network (Beggs and Plenz, 2003; Plenz and Thiagarajan, 2007). Accumulating experimental evidence further support the hypothesis that the mammalian cortex self-organizes into a critical state (Muñoz, 2018; Plenz et al., 2021). We thus analyzed the nLFP statistics in each network (Figure 5B) by calculating the branching parameter, as well as the probability distribution of avalanche sizes and durations.

Stability of the propagation can be measured by evaluating the branching parameter σ* (Priesemann et al., 2014):


(5) $$\sigma^{*}=\left\langle \sigma_{i}^{*}\right\rangle =\left\langle \text{round}\,\left(\frac{n_{i+1}}{n_{i}}\right)\right\rangle$$

where σ*_i_ is an estimate for the *i*-th bin in all avalanches of a recording, *n*_*i*_ is the number of active electrodes at *i*-th bin, round is the rounding operation to the nearest integer, and <> is the average over all *i*’s. σ*_i_ was not calculated for *n*_*i*_ = 0. Evaluation of σ* revealed that this value was slightly above 1 for both random and modular networks, suggesting a near-critical state with a tendency toward supercriticality (Figure 5C). When the network is in a critical state, neuronal activity neither increases nor decreases in avalanches and thus stably propagates in time and space. When the network is supercritical, the activity tends to expand once initiated.

The difference between the random and modular architectures was most evident in the avalanche size distribution. For a neuronal network operating near a critical state, distributions of avalanche sizes *S* and avalanche duration *T* are scale-invariant and obey a power law such that *p*(*S*)∝*S*^−τ^ and *p*(*T*)∝*T*^−α^, where τ and α are the power-law exponents. The distribution of avalanche sizes and durations averaged over the samples are shown in Figures 5D, E, respectively, for both the random and modular networks. The avalanche size distribution for the random network exhibited a deviation from the power law as a small peak near *S* = 20–40, which is an indication of supercritical dynamics. A peak was less prominent and shifted toward a smaller value of *S* in the recordings from the modular networks, which is most likely due to the segregation of the neuronal network in subpopulations. This result suggests that fabrication of structured networks with a larger number of modules may help to realize engineered neuronal networks with dynamics near criticality, along with a proper balancing of excitation and inhibition that develops with the days in culture and is a strong control parameter for avalanche dynamics (Yada et al., 2017; Muñoz, 2018; Plenz et al., 2021).

## Discussion

We described herein a feasible protocol using a cell-permissive hydrogel to interface HD-MEA surface with microfluidic devices for controlling the structure and function of cultured neuronal networks. Spontaneous activity was recorded for two types of neuronal networks, i.e., random and modular networks. The analysis of the correlation coefficient and functional complexity revealed that the network activity in the modular network was less synchronized with enriched variability compared to that in the random network. The result is in agreement with a previous report using fluorescent calcium imaging (Yamamoto et al., 2018; Takemuro et al., 2020), but the HD-MEA recording enabled a high-temporal resolution recording of the neural activity with a time step of 50 μs. Analysis of high-temporal resolution data further elucidated the dependence of the mean correlation coefficients on time bin width, which revealed that the mean correlation coefficients with and without connections between modules differ significantly as time bin widths range from 5 to 20 ms. HD-MEA recordings also enabled the analysis of LFP signals and neuronal avalanches in micropatterned neuronal networks.

Multiple methods, including laser lithography (Schürmann et al., 2018), microcontact printing (James et al., 2000; Nam et al., 2006), agarose-gel patterning (Suzuki et al., 2005; Hong and Nam, 2020), and microfluidic devices (Pan et al., 2015; Forró et al., 2018), have been used to pattern dissociated neurons on MEAs. Patterning dissociated neurons on HD-MEA has been demonstrated using microfluidic devices, firstly by Lewandowska et al. (2015) and later by Kim et al. (2020) and Duru et al. (2022). The major challenge in patterning neurons on HD-MEA devices lies in the surface topography of the device, which originates in the passivation layer and the underlying electronics, which inhibit stable sealing of the microfluidic device, as was shown in Figure 1C. Lewandowska et al. (2015) bypassed this problem by aligning the straight microchannels parallel to the surface grooves, whereas Duru et al. (2022) resolved this issue more comprehensively by establishing a protocol to bond microfluidic devices with diluted PDMS gel.

In the present work, we proposed a method to interface surface topography of the HD-MEA with PDMS microfluidic devices by coating the HD-MEA with a stable hydrogel membrane. Our method is economical and allows repeated use of an HD-MEA chip for at least three times. More importantly, this method enabled the use of sub-10 μm microchannels, which was challenging in the PDMS-gluing approach due to clogging of the channels (Duru et al., 2022). One limitation of the present approach is that chip preparation requires three additional days prior to cell seeding, whereas the PDMS gluing can be completed in several hours (Duru et al., 2022). As the present experiments adopted a previously published protocol for the preparation of the hydrogel film (Matsumura et al., 2016), it remains to be investigated whether the process can be simplified to reduce the preparation time. Another limitation is the increase in cell-electrode distance due to the insertion of the hydrogel layer. Increased cell-electrode distance inevitably decreases the signal amplitude (Figure 2) and may impede applications in recordings that demand higher signal-to-noise ratio. In these cases, other approaches may need to be considered for sealing microfluidic devices, such as the one that uses a thin layer of PDMS gel to glue microfluidic devices to the electrodes (Duru et al., 2022). We finally note that certain HD-MEAs have been developed with a more planar surface than the device used in the present research (Bertotti et al., 2014; Zeck et al., 2017), and in such a case, interface treatment might not be necessary. The approach reported herein is nevertheless important as a method to stably interface microfluidic devices to microstructured surfaces in general, such as implant biomaterials (Zhang et al., 2022).

Cell engineering based on microfluidics has become an indispensable technology for studying structure-function relationships and modeling neuronal network functions *in vitro*, and the high-temporal resolution of the MEA, along with its potential to record LFPs, enables the analysis of novel aspects of the engineered networks. In addition to network modularity, precise control of axon orientation (Peyrin et al., 2011) and even dendritic spines (Mateus et al., 2022) has been demonstrated using microfluidic devices. Combination of such neuroengineering technology with state-of-the-art MEA devices will open new application of *in vitro* systems as a tool in fundamental neuroscience and pharmacology.

## Data availability statement

The raw data supporting the conclusions of this article will be made available by the authors, without undue reservation.

## Ethics statement

The animal study was reviewed and approved by Center for Laboratory Animal Research, Tohoku University.

## Author contributions

YS conducted the experiments and analyzed the data. HY designed the research and wrote the first draft of the manuscript. HK performed the neuronal avalanche analysis. TT, SS, and AH-I jointly supervised the research. All authors contributed to the article and approved the submitted version.
