# Supplementary material for: Microfluidic cell engineering on high-density microelectrode arrays for assessing structure-function relationships in living neuronal networks
Source: Front Neurosci. 2023 Jan 9;16:943310. doi: 10.3389/fnins.2022.943310 (PMC9868575; doi:10.3389/fnins.2022.943310)
Supplement: Supplementary file 1 [file Image_1.pdf]

Supplementary Figures

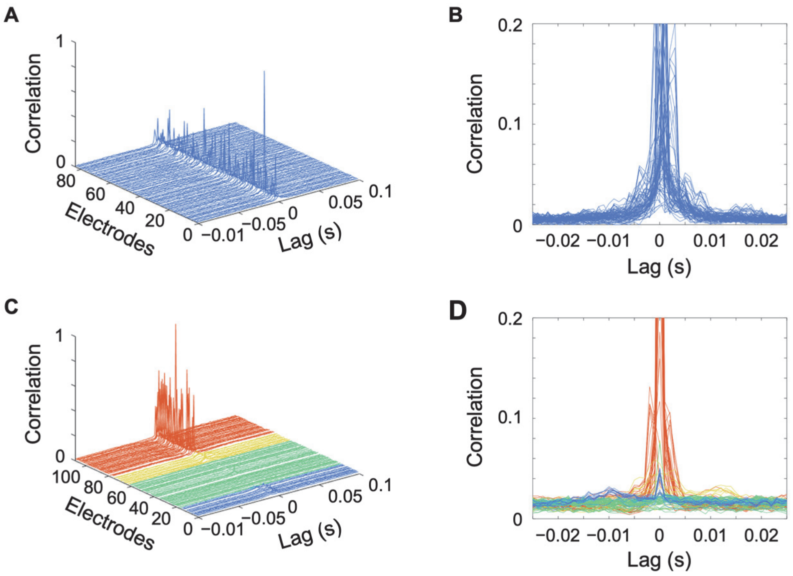

Supplementary Figure 1. Cross-correlation functions for one electrode in relation to all other electrodes in a random network **(A, B)** and a modular network **(C, D)**. Cross-correlation functions were calculated against electrode #10 and #92 for the random and modular networks, respectively, using spike trains binned at 1 ms. The same data as the ones presented in Fig. 3 are used. In panels C and D, the functions are colored according to the modules.

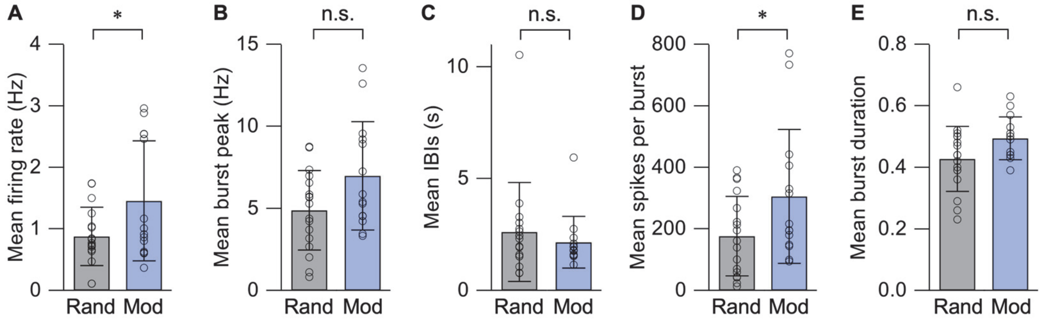

Supplementary Figure 2. Summary of activity statistics in random (Rand) and modular (Mod) networks at 10–14 DIV. **(A)** Mean firing rate, obtained by averaging the instantaneous firing rate of an electrode over all electrodes and time. (**B−F**) Burst statistics. A burst was detected when an instantaneous firing rate averaged over all electrodes increased over its root-mean-square, a condition which detects network-wide bursting events but neglects a fraction of smaller events in modular networks. A burst peak was defined as the peak firing rate of the burst **(B)**. An onset (offset) of a burst was detected as the time when the firing rate increased (decreased) the 40% value of the burst peak. An inter-burst interval (IBI) was defined as the time between the offset of a previous burst to the onset of a subsequent burst **(C)**. Spikes per burst was the total number of spikes in a burst **(D)**, and burst duration was the time between the onset and offset of a given burst (**E**). The values were averaged over the entire recording period and over all electrodes to obtain the means. Open circles represent independent networks (n = 17 and 14 for random and modular networks, respectively), bar heights the means, and error bars the SD. *$p < 0.05$; n.s., no significance (two-tailed t-test).

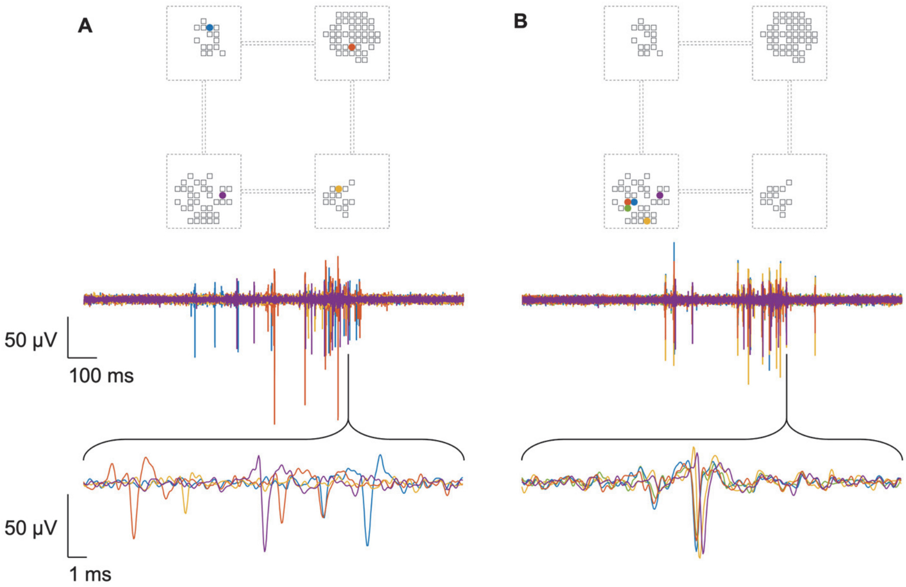

Supplementary Figure 3. Propagation of neural activity in a modular network (14 DIV). **(A)** Representative waveforms of four electrodes in four modules during a network burst. **(B)** Representative waveforms of five electrodes in a single module during a network burst. *Top*, electrode configuration. Dashed squares denote the presumptive outline of the microfluidic device, small gray squares denote the positions of active electrodes, and colored circles indicate the active electrodes whose signals are presented in the middle and bottom panels. *Middle*, waveforms from the four electrodes across the entire network burst. *Bottom*, a magnified view of the waveforms. Negative deflections in the waveforms correspond to action potentials.

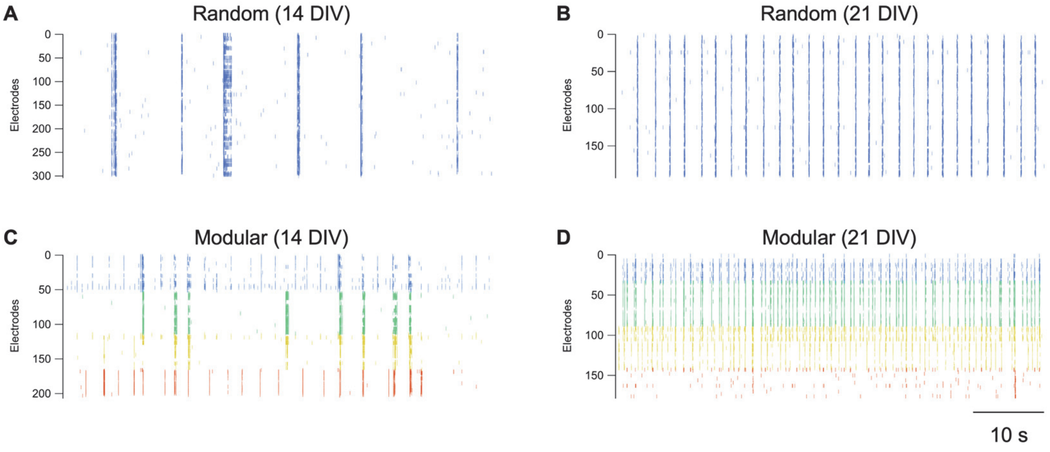

Supplementary Figure 4. Long-term culture of micropatterned primary neurons on HD-MEA. **(A, B)** Representative recordings of spontaneous activity from a random network recorded at **(A)** 14 and **(B)** 21 DIV. While network bursts were dominant in both recordings, the duration and frequency of individual bursting events decreased and increased, respectively, with culture age, suggesting the maturation of both inhibitory and excitatory interactions. **(C, D)** Same as **(A, B)** but for a modular network. Network bursts coexist with partially synchronized activity (probabilistic coherence) in modular cultures **(C)**, and such an activity mode was retained for at least up to 21 DIV **(D)**. As in the random networks, the decreased duration and increased frequency of the bursting events were observed. The number of electrodes used to record the networks varies in the recordings from 14 and 21 DIV, since active electrodes were scanned and selected for each recording.
